# Repeated whole-body inhalation of cannabidiol vapor induces progressive pulmonary inflammatory and vascular remodeling without systemic toxicity in Sprague–Dawley rats

**DOI:** 10.14202/vetworld.2026.3701-3715

**Published:** 2026-08-27

**Authors:** Chutchai Piewbang, Sitthiphon Bunman, Tanit Kasantikul, Sombat Muengtaweepongsa, Prakasit Wannapaschaiyong, Somporn Techangamsuwan

**Affiliations:** 1 Department of Pathology, Faculty of Veterinary Science, Chulalongkorn University, Bangkok 10330, Thailand.; 2 Center of Excellence in Animal Virome and Diagnostic Development, Faculty of Veterinary Science, Chulalongkorn University, Bangkok 10330, Thailand.; 3 Department of Community Medicine and Family Medicine, Faculty of Medicine, Thammasat University, Pathum Thani 12120, Thailand.; 4 Center of Excellence in Stroke, Faculty of Medicine, Thammasat University, Pathum Thani 12120, Thailand.; 5 Department of Pathobiology and Diagnostic Investigation, College of Veterinary Medicine, Michigan State University, East Lansing, Michigan 48910, USA.; 6 Department of Pediatrics, Faculty of Medicine Siriraj Hospital, Mahidol University, Bangkok 10700, Thailand.

**Keywords:** cannabidiol, inhalation toxicology, pulmonary inflammation, pulmonary vascular remodeling, rat model, respiratory safety, Sprague–Dawley rats, whole-body inhalation

## Abstract

**Background and Aim:**

Cannabidiol (CBD) is increasingly administered via electronic vaporizers because inhalation enables rapid pulmonary absorption and bypasses first-pass hepatic metabolism. However, the respiratory consequences of repeated exposure to purified CBD vapor remain insufficiently characterized. This study investigated the systemic and pulmonary effects of repeated whole-body inhalation of purified CBD vapor in Sprague–Dawley rats, with emphasis on the temporal progression of pulmonary histopathological changes.

**Materials and Methods:**

Thirty 9-week-old male Sprague–Dawley rats were randomly allocated to five groups (n = 6/group): saline-exposed control and CBD vapor exposure for 15, 30, 60, or 90 consecutive days. Approximately 100 mg of purified CBD isolate was loaded into an electronic vaporization device for each exposure session, and individual rats underwent whole-body exposure for 5 min/day under standardized chamber conditions. Clinical status, body weight, and rectal temperature were monitored. Hematological, biochemical, and electrolyte parameters were measured at the end of each exposure period. Lung and extrapulmonary tissues were examined histopathologically. Pulmonary lesions were independently evaluated by two blinded veterinary pathologists using a semi-quantitative grading scale.

**Results:**

No mortality, clinically apparent morbidity, abnormal respiratory signs, or significant differences in body weight were observed. Several hematological and biochemical variables differed significantly among groups; however, most post hoc comparisons were nonsignificant, and all values remained within physiological reference intervals. No histopathological abnormalities were detected in the liver, gastrointestinal tract, kidneys, or brain. In contrast, repeated CBD vapor exposure produced progressive pulmonary inflammatory and vascular alterations. Interstitial pneumonia and bronchus-associated lymphoid tissue hyperplasia were evident from 15 days, whereas perivascular eosinophilic cuffing and pulmonary arterial smooth muscle hypertrophy increased with exposure duration. The most severe lesions occurred after 60 and 90 days and included marked interstitial inflammation, dense eosinophilic perivascular infiltrates, hemorrhagic foci, and advanced arterial remodeling.

**Conclusion:**

Repeated whole-body inhalation of purified CBD vapor was associated with progressive, localized pulmonary inflammatory and vascular changes despite the absence of clinically meaningful systemic toxicity. These findings support further studies incorporating aerosol characterization, inhaled dosimetry, pulmonary function testing, mechanistic biomarkers, recovery assessment, and clinically relevant vaping formulations.

## INTRODUCTION

Cannabidiol (CBD) is a major non-psychoactive phytocannabinoid derived from *Cannabis sativa* that has attracted considerable scientific and clinical interest because of its broad therapeutic potential. Unlike Δ9-tetrahydrocannabinol (THC), the principal psychoactive constituent of cannabis, CBD exhibits minimal affinity for cannabinoid receptor type 1 (CB1), thereby lacking the psychotropic effects typically associated with THC [1, 2]. Instead, CBD interacts with multiple molecular targets, including cannabinoid receptor type 2 (CB2), transient receptor potential vanilloid 1 (TRPV1) channels, and serotonin 5-hydroxytryptamine 1A (5-HT1A) receptors. Through these diverse pharmacological interactions, CBD has been reported to exert anxiolytic, anti-inflammatory, antiepileptic, neuroprotective, and immunomodulatory effects [3–5].

CBD is administered through several routes, including oral, topical, sublingual, and inhalational formulations. Among these, inhalation through electronic cigarettes and vaping devices has become increasingly popular because it provides rapid systemic absorption via the pulmonary circulation while avoiding first-pass hepatic metabolism [6, 7]. Consequently, inhaled CBD produces a faster onset of action than oral formulations and has become a preferred route of administration among recreational users and individuals seeking rapid therapeutic effects [8].

The route of CBD administration substantially influences its pharmacokinetic profile and tissue-specific biological responses. Unlike orally administered CBD, inhaled CBD comes into direct contact with the respiratory epithelium before entering the systemic circulation, resulting in rapid pulmonary absorption and immediate exposure of airway tissues to aerosolized constituents [9]. Therefore, inhalational administration may elicit biological responses that differ considerably from those associated with oral or parenteral administration, particularly with respect to pulmonary safety. Given the rapidly increasing popularity of CBD vaping products, understanding their respiratory effects has become an important public health, toxicological, and regulatory priority.

Despite the widespread availability and increasing consumption of vaporized CBD products, the respiratory consequences of repeated inhalation remain inadequately characterized [10]. Most previous studies have focused on oral CBD administration, acute inhalation exposure, mixed cannabinoid preparations, nicotine-containing electronic cigarette aerosols, THC-containing cannabis products, or commercially formulated vaping liquids [8, 10–14]. Consequently, the specific pulmonary effects attributable to purified CBD vapor remain difficult to distinguish. Moreover, aerosol generation during electronic vaporization can produce ultrafine particles, reactive carbonyl compounds, and other thermal degradation products that can induce airway irritation, oxidative stress, and inflammatory responses independent of CBD [15, 16]. Therefore, pulmonary responses following CBD inhalation are likely due to complex interactions among CBD, aerosolized particles, and vaporization-derived chemical constituents.

Experimental evidence regarding the pulmonary effects of inhaled cannabinoids remains inconsistent. Although CBD has demonstrated anti-inflammatory and antifibrotic properties in several models of allergic airway inflammation and systemic inflammatory diseases [17, 18], inhalation studies using aerosolized cannabinoid formulations have also reported pulmonary inflammation, immune activation, epithelial injury, and structural lung damage [10, 19, 20]. These seemingly contradictory findings suggest that the biological effects of inhaled CBD are strongly influenced by factors such as aerosol composition, vaporization conditions, exposure duration, and route of administration. However, the long-term pulmonary consequences of repeated inhalation of purified CBD vapor remain poorly understood.

Whole-body inhalation exposure provides an experimental model that more closely simulates repeated environmental exposure during routine vaping while minimizing the restraint-associated stress inherent to prolonged nose-only exposure systems [21]. Although this exposure model does not permit precise quantification of the inhaled dose for individual animals, it enables standardized evaluation of cumulative pulmonary responses under controlled experimental conditions over extended exposure periods.

Although previous investigations have provided valuable information on the pharmacological properties and therapeutic potential of CBD, important knowledge gaps remain regarding its respiratory safety during chronic inhalation exposure. Most published studies have evaluated acute exposure, oral administration, mixed cannabinoid formulations, nicotine-containing aerosols, or commercially available vaping products, making it difficult to determine the pulmonary effects associated specifically with repeated exposure to purified CBD vapor. Furthermore, few studies have simultaneously examined the temporal progression of pulmonary histopatho-logical alterations together with hematological and biochemical indicators of systemic toxicity following prolonged whole-body inhalation. Consequently, the relationship between repeated CBD vapor exposure, progressive pulmonary tissue remodeling, and systemic safety has not been comprehensively characterized under standardized experimental conditions.

Therefore, this study aimed to investigate the systemic and pulmonary effects of repeated whole-body inhalation of purified CBD vapor in Sprague–Dawley rats following exposure periods of 15, 30, 60, and 90 days. Particular emphasis was placed on characterizing the temporal progression of pulmonary histopathological alterations, including inflammatory and vascular remodeling, while concurrently evaluating hematological and biochemical biomarkers of systemic toxicity. By integrating longitudinal histopathological assessment with systemic safety evaluation, this study sought to provide a comprehensive experimental characterization of the pulmonary effects of prolonged inhalation of purified CBD vapor and to generate evidence to inform future respiratory safety assessments of inhaled CBD products.

## MATERIALS AND METHODS

### Ethical approval

All experimental procedures involving animals were reviewed and approved by the Institutional Animal Care and Use Committee (IACUC), Thammasat University, Thailand (Approval No. 005/2024), before the commencement of the study. The experimental protocol complied with the institutional guidelines for the care and use of laboratory animals and adhered to the principles of the Animal Research: Reporting of *In Vivo* Experiments (ARRIVE) 2.0 guidelines to ensure the ethical design, conduct, reporting, and welfare of experimental animals. Throughout the study, all efforts were made to minimize animal suffering, reduce stress during handling and inhalation exposure, and use the minimum number of animals necessary to achieve the scientific objectives. Animals were monitored daily for clinical condition, behavior, body weight, and signs of distress, and predefined humane endpoint criteria were implemented in accordance with institutional animal welfare requirements. At the completion of each assigned experimental period, all rats were humanely euthanized by isoflurane overdose before tissue collection, in accordance with the American Veterinary Medical Association (AVMA) Guidelines for the Euthanasia of Animals (2020 Edition). No unexpected animal deaths or welfare-related adverse events occurred during the experimental period.

### Study period and location

The study was conducted from October 1, 2024, to August 31, 2025, at the Laboratory Animal Center, Thammasat University, Thailand. Animal housing, acclimatization, inhalation exposure, clinical monitoring, sample collection, and tissue processing were performed under standardized laboratory conditions.

### Study design

This randomized controlled experimental study evaluated the systemic and pulmonary effects of repeated whole-body inhalation of purified CBD vapor. Thirty male Sprague–Dawley rats were randomly assigned to five experimental groups (n = 6 per group): a saline-exposed control group and four groups exposed to CBD vapor daily for 15, 30, 60, or 90 consecutive days. These exposure periods were selected to characterize early responses after 15 days, progressive changes after 30 and 60 days, and the effects of prolonged repeated exposure after 90 days.

### CBD preparation

The CBD isolate used in this study was derived from *Cannabis sativa* L. and obtained from Cannex Pharma Co., Ltd. (Bangkok, Thailand). The isolate was produced through ethanol extraction and supplied as a purified phytocannabinoid preparation intended for research use. According to the manufacturer’s certificate of analysis, the CBD isolate had a purity of ≥99%. Trace impurities, including minor cannabinoids and residual solvents, were within acceptable analytical limits, as reported by the manufacturer.

The CBD material was stored under controlled laboratory conditions with appropriate temperature regulation and protection from light. All experiments were completed before the stated expiration date to maintain chemical stability. For inhalation exposure, the CBD isolate was loaded into refillable electronic cigarette cartridges designed for vaporization. The formulation contained purified CBD isolate without additional cannabinoids or THC.

### Inhalation apparatus and vapor delivery system

Controlled whole-body vapor exposure was performed using a sealed acrylic inhalation chamber measuring 26.6 × 42.5 × 18.5 cm (Lightec Inc., Tokyo, Japan). CBD vapor was generated using an electronic cigarette-based vaporization system equipped with MT32 coils with a resistance of 2.2 Ω. The system was operated at a constant output voltage of 3.7 V and fitted with a Protank 3 atomizer (Kanger Tech, Shenzhen Kanger Technology Co., Ltd., Shenzhen, China).

**Figure 1:**
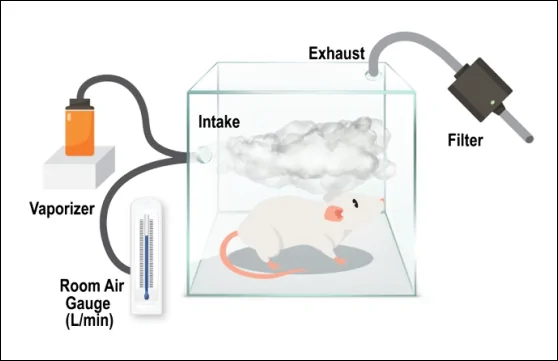
Schematic representation of the cannabidiol vapor delivery system. Vapor was generated using an apparatus adapted from electronic cigarette technology and introduced into a transparent acrylic inhalation chamber. Each exposure session lasted 5 min/day, and control animals received saline vapor under identical conditions.

During each exposure session, the vaporization device was continuously activated throughout the 5-min exposure period to generate a consistent supply of CBD-containing vapor (Figure 1). The inhalation chamber was operated under controlled airflow using balanced air intake and vacuum-assisted exhaust at approximately 1 L/min, thereby promoting continuous mixing of aerosolized CBD within the chamber. The chamber dimensions, device configuration, coil resistance, operating voltage, airflow rate, exposure duration, and amount of CBD introduced into the vaporization cartridge were maintained consistently throughout the study to minimize inter-session variability.

### Animals and housing conditions

Nine-week-old male Sprague–Dawley rats weighing 300–350 g were obtained from Nomura Siam International, Bangkok, Thailand, and housed at the Laboratory Animal Center, Thammasat University. Before study initiation, all animals were visually examined to confirm their health status.

The rats were maintained under controlled environmental conditions comprising a 12-h light/dark cycle, an ambient temperature of 22 ± 1°C, and relative humidity of 30%–70%. They were housed two animals per polycarbonate cage measuring 26.6 × 42.5 × 18.5 cm and were provided with standard laboratory chow and filtered tap water *ad libitum*. The bedding consisted of corncob and wood shavings and was replaced every 2 days. Water bottles were cleaned and replaced regularly, and all cages were maintained under standard hygienic conditions.

Environmental enrichment was provided throughout the experimental period and included hide boxes, plastic or cardboard tubes, shredded paper, wooden chew blocks, and running wheels. The animals were acclimatized for 1 week before the experiment and continued to receive standard chow and water *ad libitum* during acclimatization.

### Experimental groups and randomization

Following acclimatization, the rats were assigned to five experimental groups using a simple randomization procedure based on the duration of CBD vapor exposure. Each group contained six animals. The groups comprised a saline-exposed control group and four groups exposed to CBD vapor for 15, 30, 60, or 90 consecutive days.

The selected exposure durations were intended to characterize the time-dependent development and progression of pulmonary lesions associated with repeated inhalation of CBD vapor. The 15-day period represented an early response; the 30- and 60-day periods represented progressive changes during continued exposure; and the 90-day period represented the consequences of prolonged, repeated exposure.

### CBD vapor exposure protocol

Approximately 100 mg of CBD isolate was introduced into the vaporization device before each exposure session. During each session, each rat was placed individually inside the inhalation chamber and exposed to the generated CBD-containing vapor for 5 min. The vaporization device remained continuously activated throughout the exposure period under standardized operating conditions.

After each exposure, the chamber was cleaned with 70% ethanol, rinsed with distilled water, and allowed to dry completely before introducing the next animal to minimize cross-contamination. The saline-exposed control group was maintained under corresponding exposure conditions to establish baseline physiological and histopathological parameters.

All exposure sessions were conducted in the morning at approximately the same time each day to minimize potential circadian variability. During each exposure session, the animals were continuously observed for alterations in general behavior and signs of respiratory distress. The chamber was operated under ventilated conditions to maintain adequate air exchange throughout the exposure period.

Throughout the experiment, the animals were monitored daily for general health status, body weight, food and water intake, behavior, respiratory distress, and other abnormal clinical conditions.

### Clinical monitoring

The general clinical condition of each rat was evaluated twice daily throughout the experimental period, immediately before and after each exposure session. Assessments included body condition, external appearance, respiratory pattern, grooming behavior, locomotor activity, responsiveness to environmental stimuli, and the presence of nasal or ocular discharge, crusting, or porphyrin staining.

Animals exhibiting abnormal clinical signs during the acclimatization period were excluded prior to study initiation. Animals that developed severe illness or met predefined humane endpoint criteria in accordance with institutional animal welfare guidelines were to be humanely euthanized and excluded from further study. However, no animals met these criteria during the experimental period.

Body weight was recorded at baseline and every 2 weeks thereafter using an animal weighing scale (Model R21PE, OHAUS Corporation, Parsippany, NJ, USA). Rectal body temperature was measured using a digital thermometer. Baseline temperature was recorded immediately before each inhalation session, whereas post-exposure temperatures were measured at 1, 2, 3, and 4 h after completion of the exposure session to assess potential acute physiological responses to CBD vapor inhalation.

### Blood collection

At the end of each assigned exposure period, the animals were anesthetized with 2% isoflurane and euthanized by terminal cardiac puncture. Whole-blood samples intended for hematological analysis were collected into ethylenediaminetetraacetic acid-containing tubes, whereas samples intended for biochemical analysis were collected into appropriate tubes and allowed to coagulate before serum separation.

### Hematological analysis

Whole-blood samples were analyzed using an automated veterinary hematology analyzer (ABX Micros ESV 60, Horiba ABX, Montpellier, France). The evaluated hematological parameters included white blood cells (WBC) count, red blood cells (RBC) count, hemoglobin (HGB) concentration, hematocrit (HCT), and platelet (PLT) count.

### Serum biochemical analysis

Serum was separated from coagulated blood samples and analyzed using an automated clinical chemistry analyzer (Dri-Chem NX500, FUJIFILM Corporation, Tokyo, Japan). Renal function was assessed by measuring blood urea nitrogen (BUN) and creatinine concentrations. Hepatic integrity and enzyme activity were evaluated by measuring alanine aminotransferase (ALT), aspartate aminotransferase (AST), and alkaline phosphatase (ALP). Albumin (ALB) and plasma protein concentrations were assessed as indicators of protein status and hepatic synthetic function. Electrolyte homeostasis was evaluated by measuring sodium (Na⁺), potassium (K⁺), and chloride (Cl⁻) concentrations.

Plasma protein concentration was measured using a colorimetric Bradford protein assay kit (Bio-Rad Laboratories, Hercules, CA, USA), with bovine serum ALB (Amresco LLC, Solon, OH, USA) as the standard. Absorbance was measured at 595 nm using an iMark™ microplate absorbance reader (Bio-Rad Laboratories).

The biochemical biomarkers were selected to evaluate potential systemic toxicity associated with repeated CBD vapor exposure. ALT, AST, and ALP were assessed as indicators of hepatic integrity; BUN and creatinine were assessed as indicators of renal function; ALB and plasma protein were evaluated as indicators of hepatic synthetic and protein status; and Na⁺, K⁺, and Cl⁻ were assessed as indicators of electrolyte homeostasis. These variables are commonly used in experimental toxicology studies to identify evidence of systemic organ dysfunction.

### Tissue collection and histological processing

Samples of the lungs, liver, gastrointestinal tract, kidneys, and brain were collected after euthanasia. The gastrointestinal samples included the duodenum, jejunum, ileum, and colon. All tissue samples were fixed in 4% paraformaldehyde for 24 h and subsequently processed using an automated tissue processor.

The processed samples were embedded in paraffin, sectioned at 3 μm, and stained with hematoxylin and eosin (H&E) for histopathological examination. Histological slides were digitized using a 3DHISTECH whole-slide scanner (3DHISTECH Kft, Budapest, Hungary).

### Histopathological evaluation

Histopathological examinations were conducted at low magnifications of 40×–100× and high magnifications of 200×–400×, as appropriate for evaluating tissue architecture and cellular morphology. For pulmonary histopathology, four lung sections were examined from each animal. These comprised sections from the cranial and caudal lobes of both the left and right lungs.

Each lung section was examined in its entirety by two board-certified veterinary pathologists who were blinded to the experimental groups. Extrapulmonary tissues, including the liver, gastrointestinal tract, kidneys, and brain, were also examined for evidence of histopathological abnormalities.

### Semi-quantitative grading of pulmonary lesions

Pulmonary lesions were selected for semi-quantitative evaluation based on their biological relevance as indicators of inhalation-associated pulmonary injury. Interstitial pneumonia was evaluated as the primary indicator of inflammatory injury within the pulmonary parenchyma. Bronchus-associated lymphoid tissue (BALT) hyperplasia was assessed as an indicator of chronic local immune activation following repeated airway exposure.

Perivascular eosinophilic cuffing was evaluated because eosinophilic infiltration may indicate hyper-sensitivity-associated or chronic inflammatory responses induced by inhaled irritants. Pulmonary arterial smooth muscle hypertrophy was assessed as an indicator of pulmonary vascular remodeling associated with persistent inflammatory injury.

The overall severity of each lesion across all examined lung sections was graded using a semi-quantitative scale of 0–3, where 0 = absent, 1 = mild, 2 = moderate, and 3 = severe. Separate scores were assigned for interstitial pneumonia, BALT hyperplasia, perivascular eosinophilic cuffing, and pulmonary arterial smooth muscle hypertrophy. Individual lesion scores were recorded for each animal and summarized as mean ± standard deviation (SD) for each experimental group.

### Statistical analysis

Body weight, body temperature, hematological variables, biochemical variables, electrolyte concentrations, and pulmonary lesion scores were summarized as mean ± SD. Statistical analyses were performed using GraphPad Prism version 9.0 (GraphPad Software, San Diego, CA, USA).

The homogeneity of variances was evaluated using the Brown–Forsythe and Bartlett tests before one-way analysis of variance (ANOVA). When the overall ANOVA demonstrated a statistically significant difference, Tukey’s multiple-comparison test was performed to identify pairwise differences among the exposure groups. ANOVA F statistics, exact p values, and 95% confidence intervals from the post hoc analyses were reported where applicable. Statistical significance was established at p < 0.05.

## RESULTS

### General clinical appearance

No mortality, clinically apparent morbidity, premature euthanasia, or animal exclusions occurred during the study, and all animals completed their assigned exposure periods. Standard laboratory chow and drinking water were provided *ad libitum* throughout the experiment. However, food and water intake were not quantitatively measured and, therefore, could not be compared among the groups. All animals tolerated the daily inhalation procedures without observable adverse clinical signs during or immediately after exposure.

Rats in the CBD-exposed and control groups maintained a healthy external appearance throughout the study. They exhibited well-proportioned bodies, smooth and glossy coats, and bright eyes without discharge. The ears were clean and free of lesions, and the nasal planum remained moist without crusting or porphyrin staining. The whiskers were symmetrical and intact. Normal behaviors, including alertness, curiosity, responsiveness to the environment, coordinated movement, and regular grooming, were consistently observed in all groups. Respiratory rates remained within the expected range of approximately 100–150 breaths/min.

**Figure 2:**
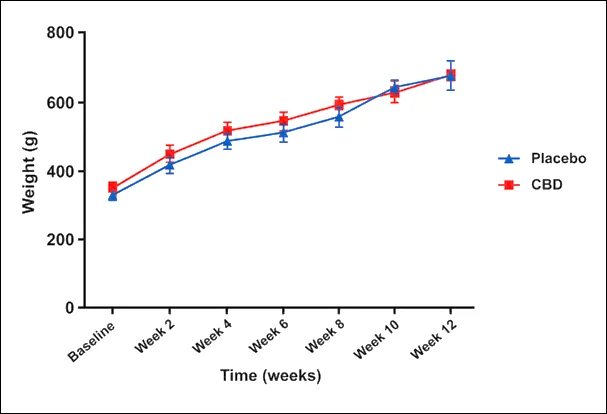
Comparison of body weight between control rats exposed to saline vapor and rats exposed to cannabidiol vapor during the 12-week experimental period. Data are presented as mean ± standard deviation (n = 6/group). No statistically significant differences in body weight were observed between the groups throughout the study.

**Figure 3:**
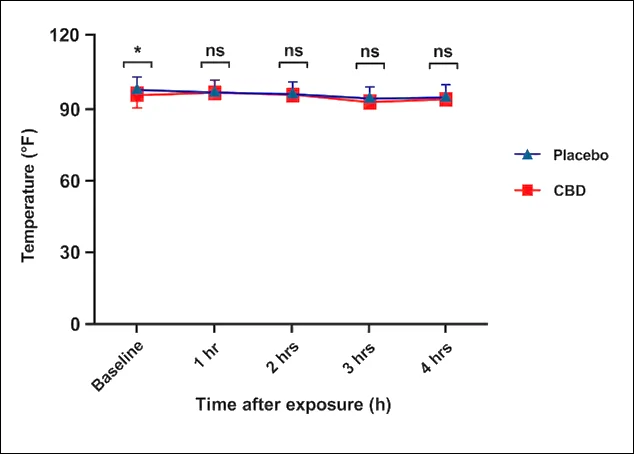
Rectal body temperature of control rats exposed to saline vapor and rats exposed to cannabidiol vapor measured at baseline and 1, 2, 3, and 4 h after exposure. Data are presented as mean ± standard deviation (n = 6/group). Statistical comparisons between the groups were performed at each time point. p < 0.05; not significant.

Body weight remained relatively stable throughout the experimental period, with no statistically significant differences between the control and CBD-exposed groups at any evaluation time point (Figure 2; Supplementary Table S1). Rectal temperature also remained stable during the post-exposure observation period. The mean baseline temperatures were 99.83 ± 0.92°F in the control group and 98.36 ± 0.69°F in the CBD-exposed group, with a statistically significant difference observed before exposure (p = 0.012). However, no significant differences were detected between the groups at 1 h (p = 0.911), 2 h (p = 0.715), 3 h (p = 0.090), or 4 h (p = 0.923) after exposure (Figure 3; Supplementary Table S2). Overall, no apparent differences in general clinical condition or external appearance were observed between the CBD-exposed and control animals.

### Hematology

Hematological analysis demonstrated significant overall differences among the experimental groups in RBC count (p < 0.0001) and HGB concentration (p = 0.0154), whereas WBC count, HCT, and PLT count did not differ significantly (Table 1). Tukey’s post hoc analysis revealed a significantly lower RBC count after 15 days of CBD exposure (p < 0.01) and a significantly higher RBC count after 90 days of exposure (p < 0.05) compared with the control group. Although the overall ANOVA indicated a significant difference in HGB concentration, no significant pairwise differences were detected after adjustment for multiple comparisons. All hematological values remained within published physiological reference intervals for healthy adult Sprague–Dawley rats.

### Biochemical parameters

Biochemical analysis demonstrated significant overall differences among the exposure groups in plasma protein (p = 0.0016), creatinine (p = 0.0241), BUN (p = 0.0074), ALP (p = 0.0059), Na⁺ (p = 0.0225), K⁺ (p < 0.0001), and Cl⁻ (p = 0.0286). In contrast, ALB, AST, and ALT did not differ significantly among the groups (Table 1).

Tukey’s post hoc analysis identified significantly higher K⁺ concentrations after 15 days (p < 0.01) and 30 days (p < 0.05) of CBD exposure compared with the control group. No significant pairwise differences were observed for the remaining biochemical parameters after adjustment for multiple comparisons. Importantly, all biochemical values remained within established physiological reference intervals, and no histopathological evidence of hepatic or renal injury was observed.

**Table 1:** Hematological and biochemical parameters of rats following different durations of cannabidiol vapor inhalation.

**Parameter**	**Control (0 days)**	**15 days**	**30 days**	**60 days**	**90 days**	Reference interval*	**ANOVA p-value**
Hematology							
WBC (×10³ cells/mm³)	10.93 ± 2.11	9.18 ± 0.92	10.10 ± 1.51	8.83 ± 0.67	8.71 ± 1.53	6.6–18.4	0.0633
RBC (×10⁶ cells/mm³)	7.91 ± 0.28	**7.05 ± 0.21‡**	8.04 ± 0.21	7.94 ± 0.30	**8.44 ± 0.30†**	6.8–9.7	**<0.0001**
HGB (g/dL)	15.28 ± 0.49	15.10 ± 0.17	15.68 ± 0.60	14.70 ± 0.34	14.78 ± 0.79	13.0–18.0	**0.0154**
HCT (%)	44.33 ± 0.51	42.83 ± 0.75	46.50 ± 2.58	44.67 ± 1.21	40.50 ± 7.20	39–53	0.0697
PLT (×10³ cells/mm³)	645.20 ± 111.60	683.70 ± 36.35	604.80 ± 68.25	601.50 ± 48.83	614.20 ± 35.76	500–1300	0.1975
Biochemistry							
Plasma protein (g/dL)	5.83 ± 0.58	5.58 ± 0.20	5.50 ± 0.00	5.83 ± 0.25	6.16 ± 0.40	5.6–7.6	**0.0016**
Creatinine (mg/dL)	0.25 ± 0.03	0.29 ± 0.01	0.25 ± 0.03	0.24 ± 0.02	0.29 ± 0.02	0.2–0.8	**0.0241**
BUN (mg/dL)	19.48 ± 1.15	17.15 ± 2.03	18.27 ± 1.15	20.72 ± 1.52	19.93 ± 1.81	10–25	**0.0074**
ALB (g/dL)	3.46 ± 0.37	3.68 ± 0.17	3.65 ± 0.10	3.53 ± 0.15	3.41 ± 0.27	3.3–4.5	0.2579
AST (U/L)	70.83 ± 9.39	78.33 ± 11.48	79.00 ± 16.05	70.17 ± 11.96	69.83 ± 16.95	50–150	0.5969
ALT (U/L)	23.33 ± 2.50	21.17 ± 2.71	20.83 ± 2.40	19.67 ± 2.50	21.83 ± 3.37	20–80	0.2386
ALP (U/L)	269.00 ± 35.43	293.20 ± 60.25	212.20 ± 21.78	228.80 ± 35.90	205.50 ± 50.56	150–450	**0.0059**
Sodium (mEq/L)	139.70 ± 0.51	137.20 ± 4.07	138.50 ± 0.83	140.30 ± 1.36	141.00 ± 0.89	135–145	**0.0225**
Potassium (mEq/L)	4.70 ± 0.27	**6.78 ± 0.27‡**	**6.13 ± 1.06†**	4.96 ± 0.50	4.56 ± 0.25	3.8–6.5	**<0.0001**
Chloride (mEq/L)	98.00 ± 1.14	96.63 ± 3.06	96.67 ± 2.87	99.00 ± 0.63	100.30 ± 1.36	95–105	**0.0286**

*Reference intervals = Representative values for healthy adult male Sprague–Dawley rats compiled from laboratory animal clinical pathology references (Petterino and Argentino-Storino, 2006; Charles River Laboratories). Data are presented as mean ± standard deviation (SD; n = 6/group). Overall differences among groups were evaluated using one-way analysis of variance (ANOVA), followed by Tukey’s multiple-comparison test. The final column reports the overall ANOVA p-value. †p < 0.05 and ‡p < 0.01 compared with the control group according to Tukey’s post hoc test. Bold ANOVA p-values indicate statistically significant overall differences among groups (p < 0.05). WBC = White blood cells; RBC = Red blood cells; HGB = Hemoglobin; HCT = Hematocrit; PLT = Platelets; BUN = Blood urea nitrogen; ALB = Albumin; AST = Aspartate aminotransferase; ALT = Alanine aminotransferase; ALP = Alkaline phosphatase.

### Statistical outcomes

One-way ANOVA demonstrated statistically significant differences among the exposure groups in RBC count (F₄.₂₅ = 21.63, p < 0.0001), HGB concentration (F₄.₂₅ = 3.781, p = 0.0154), plasma protein concentration (F₄.₂₅ = 5.976, p = 0.0016), creatinine concentration (F₄.₂₅ = 3.385, p = 0.0241), BUN concentration (F₄.₂₅ = 4.453, p = 0.0074), ALP activity (F₄.₂₅ = 4.679, p = 0.0059), Na⁺ concentration (F₄.₂₅ = 3.445, p = 0.0225), K⁺ concentration (F₄.₂₅ = 17.93, p < 0.0001), and Cl⁻ concentration (F₄.₂₅ = 3.234, p = 0.0286). WBC count, HCT, PLT count, ALB, AST, and ALT did not differ significantly among the groups (p > 0.05).

Subsequent Tukey’s multiple-comparison test identified significant pairwise differences only in RBC count after 15 and 90 days of exposure and K⁺ concentration after 15 and 30 days of exposure compared with the control group. All measured hematological and biochemical values remained within published physiological reference intervals for healthy adult Sprague–Dawley rats.

### Histopathological findings

**Figure 4:**
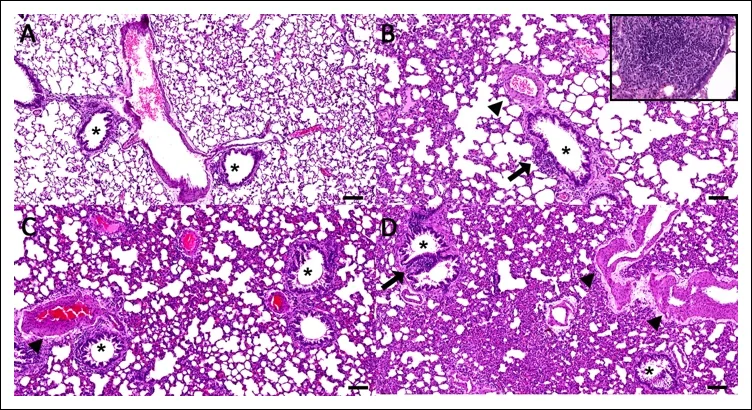
Histopathological changes in rat lungs following experimental cannabidiol vapor inhalation. (A) Control group exposed to saline vapor showing unremarkable alveolar and bronchial architecture (asterisk). (B) At 15 days, the pulmonary interstitium exhibits mild-to-moderate hypercellularity with inflammatory cell infiltration. Mild BALT hyperplasia is visible (arrow, inset), together with minimal pulmonary arterial smooth muscle hypertrophy (arrowhead). (C) At 30 days, interstitial pneumonia becomes more evident, with increased interstitial cellularity and mild vascular hypertrophy (arrowhead). (D) At 60 days, interstitial pneumonia is more pronounced, accompanied by Bronchus-associated lymphoid tissue hyperplasia (arrow) and moderate vascular hypertrophy (arrowheads). Hematoxylin and eosin stain. Asterisks indicate bronchi. Scale bars = 100 μm.

Histopathological examination of the lungs revealed a progressive pattern of inflammatory and vascular remodeling associated with the duration of CBD vapor exposure. Control animals consistently displayed normal pulmonary architecture without detectable lesions (Figure 4A).

**Figure 5:**
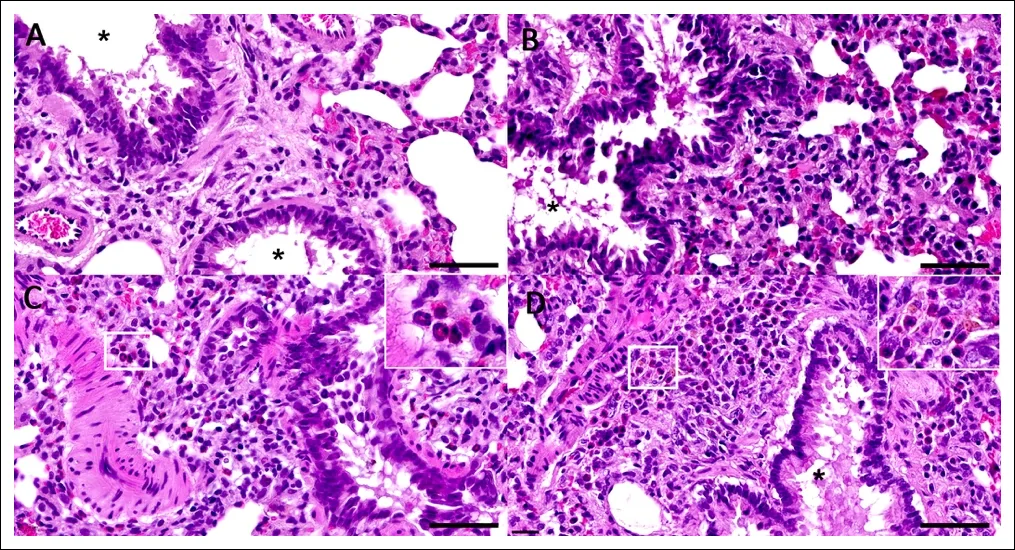
Histopathological changes in rat lungs following experimental cannabidiol vapor inhalation at 15 days (A), 30 days (B), 60 days (C), and 90 days (D). (A) Mild-to-moderate thickening of the alveolar septa with infiltration of foamy macrophages. (B) Hypercellularity becomes more pronounced, with increased numbers of foamy macrophages within the pulmonary interstitium. (C) Prominent inflammatory cell infiltration, including numerous eosinophils surrounding pulmonary vessels (inset). (D) Progressive accumulation of foamy macrophages and eosinophils around pulmonary vessels, accompanied by hemosiderin-laden macrophages. Hematoxylin and eosin stain. Asterisks indicate bronchi. Scale bars = 50 μm.

After 15 days of exposure, the lungs exhibited mild-to-moderate interstitial pneumonia characterized by diffuse thickening of the alveolar septa, infiltration by foamy macrophages, and scattered hematopoietic precursor cells (Figure 4B). BALT hyperplasia was evident and was accompanied by minimal perivascular eosinophilic cuffing and rare pulmonary arterial smooth muscle hypertrophy (Figure 5A). Occasional foci of hemorrhage and hemosiderin-laden macrophages were also observed.

After 30 days of exposure, the inflammatory response remained moderate but exhibited greater organization. The pulmonary interstitium showed increased cellularity, with infiltrates predominantly comprising foamy macrophages, eosinophils, and myeloid precursor cells (Figure 4C). Vascular alterations became more prominent, with perivascular eosinophilic cuffing occurring more frequently and at moderate severity (Figure 5B). Pulmonary arterial smooth muscle hypertrophy progressed from mild-to-moderate, whereas BALT hyperplasia persisted but was less prominent than that observed after 15 days of exposure (Figure 4C).

After 60 days of exposure, pulmonary lesion severity increased further. Interstitial thickening and cellular infiltration extended around the bronchiolar and vascular structures (Figure 4D). Multifocal perivascular eosinophilic cuffs became prominent (Figure 5C), and small areas of alveolar hemorrhage were observed, indicating continued vascular injury. Although BALT hyperplasia did not increase substantially beyond that observed at earlier time points, pulmonary arterial remodeling progressed, with moderate-to-severe smooth muscle hypertrophy.

After 90 days of exposure, pulmonary histopathological alterations reached their greatest severity. Interstitial pneumonia was consistently present, characterized by marked thickening of the alveolar walls and extensive accumulation of foamy histiocytes. Dense inflammatory infiltrates surrounded pulmonary vessels and included marked eosinophilic cuffing and granulomatous foci containing eosinophilic crystals intermingled with areas of hemorrhage. Pulmonary arteries exhibited moderate-to-marked smooth muscle hypertrophy, indicating progressive vascular remodeling after prolonged CBD vapor exposure.

Despite pronounced pulmonary alterations, no histopathological abnormalities were identified in the extrapulmonary organs, including the liver, gastrointestinal tract, kidneys, and brain, across all exposure durations. A semi-quantitative summary of pulmonary lesion grades across the exposure periods is presented in Table 2.

**Table 2:** Semi-quantitative grading of pulmonary histopathological lesions in rats following cannabidiol vapor inhalation.

**Exposure duration**	**Interstitial pneumonia**	**BALT** **hyperplasia**	**Perivascular eosinophilic ** **cuffing**	**Pulmonary arterial** **smooth muscle hypertrophy**
Control	0.00 ± 0.00 (0/6)	0.00 ± 0.00 (0/6)	0.00 ± 0.00 (0/6)	0.00 ± 0.00 (0/6)
15 days	2.83 ± 0.41 (6/6)	2.67 ± 0.52 (6/6)	1.00 ± 0.00 (6/6)	0.00 ± 0.00 (0/6)
30 days	2.83 ± 0.41 (6/6)	2.33 ± 0.52 (6/6)	1.67 ± 0.52 (6/6)	0.33 ± 0.52 (2/6)
60 days	3.00 ± 0.00 (6/6)	2.00 ± 0.00 (6/6)	2.83 ± 0.41 (6/6)	1.50 ± 0.84 (6/6)
90 days	3.00 ± 0.00 (6/6)	1.83 ± 0.41 (6/6)	3.00 ± 0.00 (6/6)	1.83 ± 0.98 (6/6)

*Pulmonary lesions were scored individually for each animal using a semi-quantitative scale (0 = none, 1 = mild, 2 = moderate, 3 = severe). Values are presented as mean ± standard deviation (SD). Numbers in parentheses indicate the incidence of affected animals/group (n = 6). BALT = Bronchus-associated lymphoid tissue.

## DISCUSSION

### Pulmonary safety concern and principal findings

The increasing use of CBD vaping products contrasts with the limited evidence regarding their long-term pulmonary safety. Although previous studies have investigated acute inhalation exposure, oral CBD administration, or formulations containing mixed cannabinoids [9, 10, 12, 13, 22–26], relatively few have examined the pulmonary consequences of repeated long-term inhalation of purified CBD vapor.

In the present study, repeated whole-body exposure to vapor generated from a purified CBD preparation for up to 90 days induced progressive pulmonary histopathological alterations, including interstitial pneumonia, BALT hyperplasia, perivascular eosinophilic cuffing, and pulmonary arterial smooth muscle hypertrophy, without evidence of clinically relevant systemic hematological or biochemical toxicity. These findings extend the current understanding of the respiratory effects of repeated inhalation of CBD vapor by demonstrating the temporal progression of pulmonary injury over prolonged exposure.

### Systemic safety and localized pulmonary responses

Although several hematological and biochemical variables showed statistically significant overall differences among the exposure groups, Tukey’s post hoc analysis identified only limited pairwise differences relative to the control group, and all measured values remained within published physiological reference intervals for healthy adult Sprague–Dawley rats. Moreover, no corresponding histopathological abnormalities were identified in the extrapulmonary organs examined.

Collectively, these findings suggest that repeated CBD vapor exposure did not produce biologically meaningful systemic toxicity under the conditions of the present study, despite the progressive pulmonary lesions observed. Although differential leukocyte counts were not performed, the absence of systemic abnormalities together with progressive pulmonary pathology suggests that repeated CBD vapor exposure may initially induce localized respiratory responses before measurable systemic effects become apparent. Because routine hematological and biochemical analyses are relatively insensitive indicators of localized pulmonary inflammation [27], future studies incorporating differential leukocyte counts, bronchoalveolar lavage fluid analysis, and inflammatory cytokine profiling would provide a more comprehensive assessment of respiratory immune responses.

### Time-dependent progression of pulmonary lesions

One of the principal findings of the present study was the progressive development of pulmonary lesions with increasing exposure duration. Compared with previous inhalation studies that primarily focused on acute exposure or commercially formulated vaping products [10, 28], the present investigation characterized the temporal progression of interstitial pneumonia, BALT hyperplasia, perivascular eosinophilic cuffing, and pulmonary arterial smooth muscle hypertrophy following repeated whole-body inhalation of purified CBD vapor.

The coexistence of inflammatory and vascular lesions, together with the absence of detectable systemic toxicity, suggests that repeated inhalation exposure may preferentially affect pulmonary tissues before producing generalized systemic effects. This longitudinal assessment provides additional insight into the progression of pulmonary injury during chronic exposure.

### Potential contribution of vaporization-derived constituents

Although purified CBD isolate was used as the starting material, the pulmonary alterations observed in this study cannot be attributed exclusively to CBD itself. Vaporization may generate ultrafine particles, reactive carbonyl compounds, and other thermal degradation products that can induce pulmonary irritation independently of the parent cannabinoid [16, 29, 30]. In addition, aerosol characteristics are influenced by device settings, heating conditions, formulation composition, and environmental factors.

Consequently, the pulmonary lesions should be interpreted as being associated with repeated exposure to CBD-containing vapor generated under the experimental conditions employed rather than as definitive evidence of direct CBD toxicity. Future investigations incorporating comprehensive aerosol characterization and quantification of the inhaled dose will be necessary to distinguish CBD's contribution from that of vaporization-derived constituents.

### Comparison with previous inhalation studies

The present findings are broadly consistent with previous experimental studies demonstrating that inhaled CBD-containing aerosols and electronic cigarette aerosols can induce pulmonary inflammation, oxidative stress, epithelial injury, and recruitment of inflammatory cells [10, 30–32]. Comparative inhalation studies have reported greater pulmonary inflammatory responses following CBD aerosol exposure than nicotine aerosol under specific experimental conditions [10, 33], whereas chronic exposure to electronic cigarette aerosols has been associated with epithelial barrier dysfunction, inflammatory signaling, and pulmonary tissue injury [34–36].

Furthermore, observations from EVALI have highlighted the importance of aerosol composition and thermal degradation products in determining pulmonary toxicity [19, 30, 32]. Although direct comparisons should be interpreted with caution due to differences in formulations, exposure conditions, and experimental designs, these studies collectively support the concept that aerosol composition, rather than CBD alone, may contribute substantially to pulmonary injury.

### Proposed mechanisms of pulmonary injury

The progressive BALT hyperplasia, perivascular eosinophilic cuffing, and pulmonary arterial smooth muscle hypertrophy observed after prolonged exposure likely reflect persistent local inflammatory stimulation of the respiratory tract. Repeated inhalation of aerosolized particles may promote epithelial injury, activation of resident alveolar macrophages, recruitment of inflammatory cells, and endothelial dysfunction, thereby resulting in chronic inflammatory signaling and early vascular remodeling [37–39]. Similar histopathological features have been reported in experimental models of chronic inhalational lung injury and hypersensitivity-associated pulmonary inflammation [40, 41]. Nevertheless, because inflammatory cytokines, oxidative stress biomarkers, BAL fluid analysis, immunohistochemistry, and molecular investigations were beyond the scope of the present study, these proposed mechanisms should be considered biologically plausible hypotheses rather than conclusions directly supported by the present findings.

### Functional significance of pulmonary lesions

Although the histopathological findings demonstrated progressive structural alterations within the lungs, their physiological significance remains uncertain because pulmonary function testing, respiratory mechanics, arterial blood gas analysis, and oxygen saturation measurements were not performed. Histopathological abnormalities do not necessarily correlate directly with measurable respiratory dysfunction, particularly during the early stages of pulmonary injury. Future investigations integrating structural, functional, and molecular assessments will therefore be important for determining the clinical relevance of the pulmonary lesions identified in the present study.

### Translational relevance of the experimental model

The present inhalation model provides valuable information regarding the pulmonary effects of repeated CBD vapor exposure but does not fully replicate human CBD vaping practices. Human exposure is highly variable and is influenced by device characteristics, puff duration, inhalation behavior, aerosol composition, and the use of commercially formulated products containing propylene glycol, vegetable glycerin, flavoring agents, and other additives [16, 29, 32, 36]. In contrast, the present study employed standardized exposure conditions using purified CBD isolate to minimize experimental variability. Accordingly, the findings should be interpreted as demonstrating pulmonary alterations associated with repeated exposure to vapor generated by purified CBD under controlled laboratory conditions, rather than as directly predicting the respiratory health effects of commercially available CBD vaping products.

### Study limitations and future directions

Several limitations should be considered when interpreting the present findings. Aerosol particle size distribution (mass median aerodynamic diameter [MMAD]), chamber aerosol concentration, vaporization temperature, aerosol stability, and the actual inhaled CBD dose were not quantified. Consequently, exposure estimates were based on the standardized amount of CBD introduced into the vaporization device rather than the dose deposited within the respiratory tract. In addition, only a single CBD exposure concentration was evaluated, precluding dose-response assessment. The relatively small sample size, absence of an *a priori* power analysis, and exclusive use of male rats may further limit the generalizability of the findings.

Additional limitations include the absence of BAL fluid analysis, inflammatory cytokine profiling, assessment of oxidative stress biomarkers, immunohistochemistry, pharmacokinetic evaluation, pulmonary function testing, recovery studies, and molecular analyses. Although histopathological evaluation was performed independently by two board-certified veterinary pathologists using predefined scoring criteria, formal inter-observer agreement analysis was not conducted. Furthermore, saline was used as the control aerosol rather than propylene glycol/vegetable glycerin-based formulations commonly used in commercial CBD vaping products. Future studies incorporating comprehensive aerosol characterization, clinically relevant vaping formulations, mechanistic investigations, pharmacokinetic evaluation, pulmonary function assessment, recovery experiments, and multiple exposure concentrations will provide a more in-depth understanding of the pulmonary effects of repeated CBD vapor exposure.

## CONCLUSION

Repeated whole-body inhalation of vapor generated from purified CBD for up to 90 days induced progressive pulmonary histopathological alterations in Sprague–Dawley rats, characterized by interstitial pneumonia, BALT hyperplasia, perivascular eosinophilic cuffing, and pulmonary arterial smooth muscle hypertrophy. In contrast, hematological and biochemical analyses remained within physiological reference intervals, and no histo-pathological abnormalities were identified in extrapulmonary organs, indicating the absence of biologically meaningful systemic toxicity under the experimental conditions. These findings suggest that repeated exposure to CBD vapor preferentially affects pulmonary tissues before detectable systemic alterations become evident.

The present study provides one of the few longitudinal experimental evaluations of repeated inhalation exposure to purified CBD vapor under standardized laboratory conditions. A major strength of this investigation is the comprehensive time-course histopathological assessment over a 90-day exposure period, which enabled characterization of the progressive nature of pulmonary injury while minimizing confounding effects associated with commercially available vaping formulations containing multiple additives. The use of independent histopathological evaluation further strengthens the reliability of the pathological observations.

From a practical perspective, these findings highlight that the absence of clinically relevant hematological or biochemical abnormalities should not be interpreted as evidence of pulmonary safety during repeated CBD vapor exposure. The progressive pulmonary lesions observed emphasize the importance of incorporating detailed respiratory histopathology, functional pulmonary assessment, and aerosol characterization into the preclinical safety evaluation of inhaled cannabinoid products. The results also support the need for regulatory safety testing that considers both the inhaled aerosol and the thermal degradation products generated during vaporization.

In conclusion, repeated inhalation of purified CBD vapor under controlled experimental conditions produced progressive localized pulmonary injury without evidence of clinically relevant systemic toxicity. These findings provide important preclinical evidence on the respiratory safety profile of chronic CBD vapor exposure and lay the foundation for future mechanistic, dose-response, aerosol characterization, and translational studies to better define the pulmonary risks associated with long-term CBD vaping.

## DATA AVAILABILITY

The datasets generated and/or analyzed during the current study are available from the corresponding author upon reasonable request.

## GENERATIVE AI DECLARATION

The authors used generative artificial intelligence (AI) (Grammarly) solely to improve the language, grammar, and readability of the manuscript. The authors carefully reviewed and edited all AI-assisted output and take full responsibility for the content of this manuscript. AI was not used to generate research data, analyze data, interpret results, or draw scientific conclusions. AI is not listed as an author.

## AUTHORS’ CONTRIBUTIONS

CP and SB: Contributed equally. CP and SB: Conceptualization, data curation, formal analysis, investigation, software, methodology, project administration, resources, supervision, validation, writing–original draft, writing–review and editing. TK: Formal analysis and investigation. PW: Investigation, methodology, supervision, visualization, writing–review and editing. SM: Investigation, methodology, supervision, visualization, writing–review and editing. ST: Supervision and writing–review and editing. All authors have read and approved the final version of the manuscript
